# Oral disease in adults treated with hemodialysis: prevalence, predictors, and association with mortality and adverse cardiovascular events: the rationale and design of the ORAL Diseases in hemodialysis (ORAL-D) study, a prospective, multinational, longitudinal, observational, cohort study

**DOI:** 10.1186/1471-2369-14-90

**Published:** 2013-04-19

**Authors:** Giovanni FM Strippoli, Suetonia C Palmer, Marinella Ruospo, Patrizia Natale, Valeria Saglimbene, Jonathan C Craig, Fabio Pellegrini, Massimo Petruzzi, Michele De Benedittis, Pauline Ford, David W Johnson, Eduardo Celia, Ruben Gelfman, Miguel R Leal, Marietta Torok, Paul Stroumza, Anna Bednarek-Skublewska, Jan Dulawa, Luc Frantzen, Juan Nin Ferrari, Domingo del Castillo, Jorgen Hegbrant, Charlotta Wollheim, Letitzia Gargano

**Affiliations:** 1 Department of Clinical Pharmacology and Epidemiology, Consorzio Mario Negri Sud, Italy; 2 Diaverum Scientific Office, Lund, Sweden; 3 School of Public Health, University of Sydney, Sydney, Australia; 4 Department of Emergency and Organ Transplantation, University of Bari, Bari, Italy; 5 University of Otago Christchurch, Addington, New Zealand; 6 Consorzio Mario Negri Sud; “Casa Sollievo della Sofferenza” Hospital, IRCCS, San Giovanni Rotondo (FG), Italy; 7 Dental clinic, University of Bari, Bari, Italy; 8 The University of Queensland, School of Dentistry, Brisbane, Australia; 9 Centre for Kidney Disease Research, University of Queensland at Princess Alexandra Hospital, Brisbane, Australia; 10 Diaverum Medical Office, Lyon, France; 11 Diaverum Medical Office, Budapest, Hungary; 12 Diaverum Medical Office, Sintra, Portugal; 13 Diaverum Medical Office, Capital Federal, Argentina; 14 Diaverum Medical Office, Poland; and Department of Nephrology, Medical University School of Lublin, Lublin, Poland; 15 Diaverum Medical Office, Poland and Department of Internal Medicine, Metabolic Diseases, Medical University of Silesia, Silesia, Poland; 16 Diaverum Medical Office, Montevideo, Uruguay; 17 Diaverum Medical Office, Madrid, Spain

**Keywords:** Chronic kidney disease, Oral disease, Periodontitis, Mortality, Prevalence

## Abstract

**Background:**

People with end-stage kidney disease treated with dialysis experience high rates of premature death that are at least 30-fold that of the general population, and have markedly impaired quality of life. Despite this, interventions that lower risk factors for mortality (including antiplatelet agents, epoetins, lipid lowering, vitamin D compounds, or dialysis dose) have not been shown to improve clinical outcomes for this population. Although mortality outcomes may be improving overall, additional modifiable determinants of health in people treated with dialysis need to be identified and evaluated.

Oral disease is highly prevalent in the general population and represents a potential and preventable cause of poor health in dialysis patients. Oral disease may be increased in patients treated with dialysis due to their lower uptake of public dental services, as well as increased malnutrition and inflammation, although available exploratory data are limited by small sample sizes and few studies evaluating links between oral health and clinical outcomes for this group, including mortality and cardiovascular disease. Recent data suggest periodontitis may be associated with mortality in dialysis patients and well-designed, larger studies are now required.

**Methods/design:**

The ORAL Diseases in hemodialysis (ORAL-D) study is a multinational, prospective (minimum follow-up 12 months) study. Participants comprise consecutive adults treated with long-term in-center hemodialysis. Between July 2010 and February 2012, we recruited 4500 dialysis patients from randomly selected outpatient dialysis clinics in Europe within a collaborative network of dialysis clinics administered by a dialysis provider, Diaverum, in Europe (France, Hungary, Italy, Poland, Portugal, and Spain) and South America (Argentina). At baseline, dental surgeons with training in periodontology systematically assessed the prevalence and characteristics of oral disease (dental, periodontal, mucosal, and salivary) in all participants. Oral hygiene habits and thirst were evaluated using self-administered questionnaires. Data for hospitalizations and mortality (total and cause-specific) according to baseline oral health status will be collected once a year until 2022.

**Discussion:**

This large study will estimate the prevalence, characteristics and correlations of oral disease and clinical outcomes (mortality and hospitalization) in adults treated with dialysis. We will further evaluate any association between periodontitis and risk of premature death in dialysis patients that has been suggested by existing research. The results from this study should provide powerful new data to guide strategies for future interventional studies for preventative and curative oral disease strategies in adults who have end-stage kidney disease.

## Background

The prevalence of chronic kidney disease (clinically-relevant structural kidney changes or urinary abnormalities, with or without reduced estimated glomerular filtration rate [below 60 ml/min per 1.73 m^2^]) [1] is increasing globally, due in part to international epidemics of obesity and diabetes mellitus. Approximately 10% to 15% of the global adult population is affected by chronic kidney disease [2-4]. In addition to an increasing prevalence, chronic kidney disease is associated with markedly impaired quality of life, sexual dysfunction, unemployment, depression, and premature mortality [5,6]. Moderate kidney disease (estimated glomerular filtration rate below 44 ml/min per 1.73 m^2^ and/or heavy proteinuria) is associated with a 2- to 3- fold increase in all-cause mortality compared with the general population and for dialysis patients the risk is much higher [7,8]. Despite poorer clinical outcomes, pharmacologic and dialysis-related interventions (including anti-platelet agents, [9] dialysis dose, [10] early dialysis initiation, [11] vitamin D compounds, [12] erythropoietins [13], phosphodiesterase-5 inhibitors, [14] or antidepressant medication [15-20]) generally do not improve clinical outcomes or quality of life, particularly for those with end-stage kidney disease treated with dialysis. Exploration of additional and modifiable determinants of health in populations with chronic kidney disease would help prioritize the evaluation of novel intervention strategies to improve clinical outcomes.

Oral disease represents a potential and preventable cause of impaired health in people with chronic kidney disease. Oral disease, including dental decay and periodontitis, affects nearly all adults in the global population [21] and is amongst one of the most costly diseases to treat for many health systems [21,22]. Chronic disease is particularly linked to poorer oral health and greater unmet dental need, including untreated dental disease, self-reported poor oral health, and tooth loss [23]. In addition, individuals who have chronic kidney disease (estimated glomerular filtration rate below 60 ml/min per 1.73 m^2^) are much less likely than the general population to attend publicly available dental care, even when controlling for age, gender, race or ethnicity, language barriers, medical insurance and income [24]. Periodontal disease is associated with cardiovascular disease in the general population [25] and emerging data suggest a link between periodontitis and mortality in people with chronic kidney disease treated with dialysis [26,27]. Oral disease is associated with inflammation [28] and malnutrition (including the protein-energy wasting syndrome) [29,30], which affect people who have chronic kidney disease disproportionately, and are considered linked risk factors for accelerated cardiovascular disease in this clinical setting (known as malnutrition, inflammation, and atherosclerosis [MIA] syndrome) [31]. The relative contributions of socioeconomic disadvantage, malnutrition and inflammation to the prevalence and outcomes of oral disease in people who have kidney disease require analysis in a large longitudinal study, ahead of potential interventional trials.

Existing data for the prevalence and severity of oral disease in chronic kidney disease patients are confined to a few studies that have small sample sizes and marked differences in the estimates of oral disease between studies that are not readily explained by study-level clinical or demographic characteristics [32]. The ORAL Diseases in hemodialysis (ORAL-D) study has been designed to survey the prevalence, severity, correlates, and outcomes of oral disease in a large consecutive population of adults with end-stage kidney disease treated with hemodialysis to assist the prioritization of future interventional research for oral disease in this population. Robust data linking oral health to relevant clinical outcomes may additionally identify the need for specific interventional trials in dialysis patients.

The ORAL-D study was specifically designed to explore the following questions:

1. Is the prevalence of oral disease higher in adults treated with hemodialysis and does the pattern of oral disease indicate/correlate with lower use of preventative dental services?

2. What are the important correlates of oral disease in hemodialysis patients including sociodemographic and clinical factors?

3. What is the prevalence of thirst and dysgeusia symptoms in adults treated with hemodialysis?

4. What are the characteristics and correlates of preventative oral habits including teeth brushing, attendance at dental care, flossing, and use of mouth wash?

5. Is there a relationship between biochemical and clinical performance measures used to evaluate quality of dialysis care and the prevalence and severity of oral disease?

6. What is the association between oral disease (dental, periodontal, salivary, or mucosal) and hospitalization or premature mortality in hemodialysis patients?

### Hypothesis

In the largest cohort study of oral disease in dialysis patients to date (the ORAL Diseases in Hemodialysis [ORAL-D] study), we will test the hypothesis that oral disease is frequently experienced by patients with end-stage kidney disease treated with hemodialysis and increases risk of total and cause-specific hospitalization and mortality when controlling for potentially confounding factors. We have evaluated oral hygiene habits and thirst in this population and whether biochemical and clinical performance measures of dialysis care are associated with increased risks of all forms of oral disease.

## Methods/design

We received ethics approval for the ORAL Diseases in Hemodialysis (ORAL-D) study from the following responsible local Human Research Ethics Committees: Comitè de Protection des Personnes Sud-Medierranèe II (France), Komisja Bioetyczna, Slaskiego Uniwersytetu Medycznego W Katowicach (Poland), CE da Diaverum Portugal (Portugal), Comite Etico de Investigacion Clinica (CEIC) de la Fundaction Puygvert and Agencia Valenciana de Salud, Departament de Salut Valencia (Spain), and Szegedi Tudomanyegyetem, Szent-Gyorgyi albert klinikai kozpont, and Regionalis human orvosbiologiai kutatasetikai bizottsaga (Hungary). Ethics approval was not required for this type of study in Italy or Argentina. All participants provided written and informed consent prior to study initiation and patient enrolment. The study is being performed in accordance with the 2000 Edinburgh, Scotland Revision of the Declaration of Helsinki, applicable ICH guidelines and Guidelines on Research Practice.

### Study design

This is a multinational, prospective (minimum 12 months’ follow-up) study, in which approximately 4500 patients with end-stage kidney disease treated with outpatient in-center hemodialysis within a collaborative network of dialysis clinics administered by a dialysis provider (Diaverum) were enrolled between July 2010 and February 2012. The clinics included in this study were from dialysis communities with heterogeneity in social and economic circumstances and for which local investigators had committed to providing good-quality data during study follow-up.

### Patient population

The study is multinational and open to all outpatient Diaverum haemodialysis treatment centers in selected countries in Europe (France, Hungary, Italy, Poland, Portugal, and Spain) and South America (Argentina).

Eligible patients have met the following inclusion criteria:

1. End-stage kidney disease

2. Currently on long-term hemodialysis for any duration

3. Aged 18 years or over

4. Treating team agreeable to patient’s involvement in the study

5. Able to provide written and informed consent

### Study procedures

Patients had met the inclusion criteria and all provided informed consent. Processes to identify and screen all potential recruits were established within each center in consultation with the ORAL-D study Steering Committee. Patient consent forms were approved by the Human Research Ethics Committee before the beginning the study. A sample consent form and patient information sheet was provided to participating sites. Participating sites filed a copy of the approved consent form and information sheet for their center with the coordinating study office. The patient had an initial consultation with study personnel to discuss study participation, which included a preliminary eligibility check. The patient was also given an information sheet for the study. If consent was provided by the patient, a copy of the signed consent form and information sheet was given to the participant. The participant gave written and informed consent before enrollment or completion of any study-specific procedures. The ORAL-D study Steering Committee monitored the medical literature and any other relevant information that might have impacted on the ongoing conduct of the study.

The study commenced on the day of enrollment. At enrollment, all participants underwent: 1) collection of demographic and clinical data including dialysis-related care; 2) completion of self-administered questionnaires on oral hygiene and thirst; and 3) comprehensive and systematic oral examination, including assessment of dental, periodontal, mucosal, and salivary characteristics (including pH and flow).

#### Demographic and clinical data

The collection of demographic, clinical/laboratory and dialysis-related data were performed by the local treating physician on standardized case report forms within one month of enrollment. Relevant data were obtained from clinical databases linked to the patient via a standardized identification code. Standardized data variables included age, gender, race, country of residence, educational, marital and occupational status, alcohol intake, smoking history, physical activity, housing, distance from dialysis unit, menopausal status, body mass index, protein catabolic rate, family income, financial stress, food intake, cause of kidney disease, diabetic status and other comorbid conditions, medication prescription, and serum parameters including hemoglobin, phosphorus, parathyroid hormone, calcium, ferritin, albumin, cholesterol, and dialysis parameters. We included specific collection of data for medications to treat depression and anxiety that may have dry mouth as a side-effect as well as oral anticoagulants to evaluate their association with oral bleeding.

#### Self-administered questionnaires

**Figure 1:**
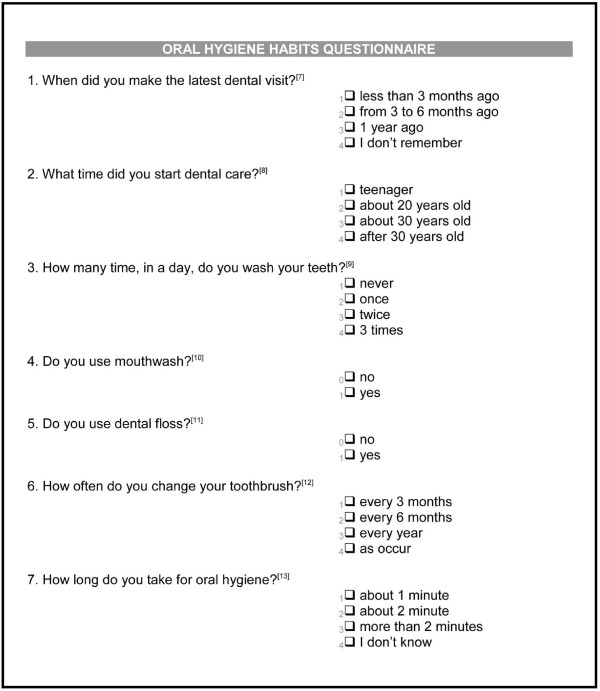
Self-administered questionnaire on oral habits.

**Figure 2:**
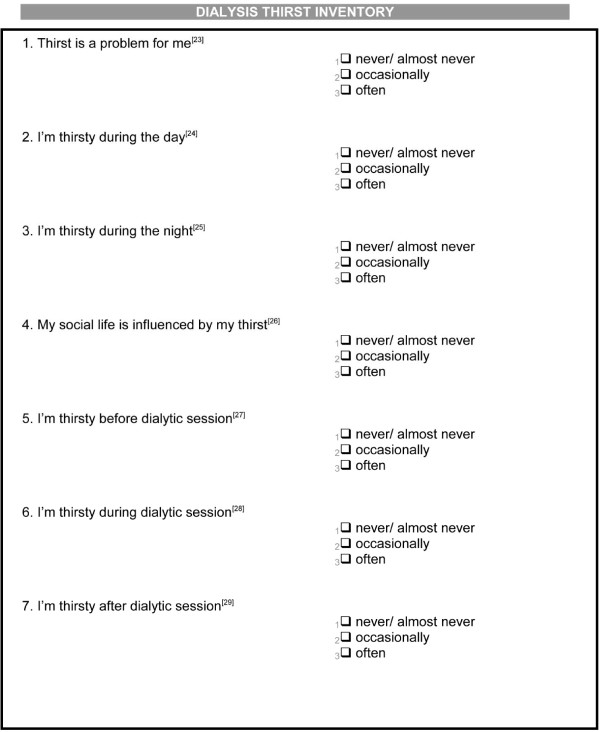
Self-administered dialysis thirst inventory questionnaire.

At baseline, all participants completed self-administered questionnaires to evaluate oral hygiene habits, [33] and thirst [34]. Oral hygiene habit data included questions about visits for dental care, brushing, mouth wash use, and dental floss practices, and duration of oral hygiene each day (Figure 1). The dialysis thirst inventory was used to ask patients about thirst as a clinical symptom including at night, the effect of social life on thirst, and thirst in relation to dialysis (Figure 2).

#### Oral examination

All patients were examined at baseline by a qualified dental surgeon with training in periodontology in each country who had been centrally trained on the protocol and methods used for the study. The standardized oral examination included analysis of dental, periodontal, mucosal and salivary health. The oral examination occurred in the dialysis unit on the day of dialysis. We performed the dental and periodontal examination before dialysis treatment started (to minimize excessive bleeding due to heparin administration). We collected saliva before and after dialysis therapy and participants completed all questionnaires during dialysis. We performed oral visits in a specifically set up room at the dialysis clinical with an appropriate chair and mobile light source, or in the dialysis room using a light-emitting diode (LED) headlamp, based on patient preference. We used a standard sterile oral examination kit containing a dental examination mirror, ball-tip periodontal probe (calibrated in millimeters), tweezers, masks, bib, tissue wipes, cotton rolls and a biodegradable tray.

1. Dental examination

The DMFT index quantifies dental caries, fillings, and tooth loss. In this study, the DMFT index was calculated centrally for 32 teeth in the adult dentition including the four wisdom teeth or third molars. The dentist also recorded the total number of teeth (maximum 32) and the presence or absence of enamel hypoplasia and dental attrition or erosion. If dental attrition or erosion was present, the dentist indicated the likely cause. The dentist also recorded the presence of any denture (partial or total).

2. Periodontal examination

**Figure 3:**
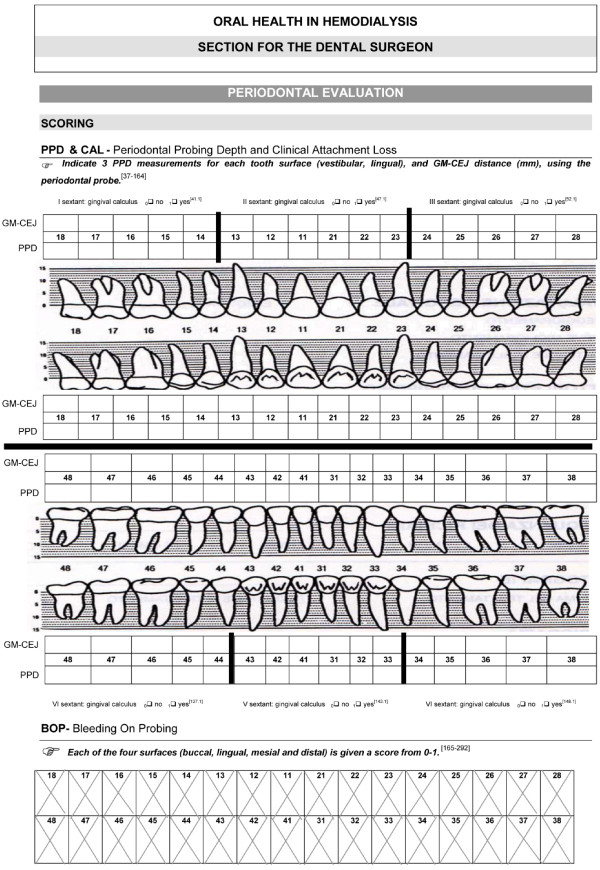
Standardized periodontal evaluation at baseline.

The standardized periodontal examination included the Periodontal Probing Depth (PPD), the Clinical Attachment Loss (CAL) score and the Bleeding on Probing (BOP) indices (Figure 3).

The Periodontal Probing Depth (PPD) was the distance between the free gingival margin and the apical extent of the periodontal pocket. The tip of the periodontal probe was placed with light pressure (10 to 20 grams) into the gingival sulcus, which is an area of potential space between a tooth and the surrounding tissue. The first marking visible above the pocket indicated the measurement of the pocket depth. Three PPD measurements for each of the vestibular and lingual aspects of the tooth were recorded. A healthy pocket depth is approximately 3 mm without bleeding on probing. Depths greater than 3 mm are associated with “attachment loss” of the tooth to the surrounding alveolar bone, characteristic of periodontitis. The PPD score was calculated as a mean value expressed in millimeters and determined as the total PPD divided by the number of sites examined.

The same periodontal probe was used to measure the distance between the cementum-enamel junction (CEJ) and the free gingival margin (GM). Clinical Attachment Loss (CAL) was calculated as the sum of these distances, according to the standard formula CAL = PPD + (GM – CEJ). The CAL score for each patient was calculated as the sum of the mean PPD (sum of all values divided by the number of sites examined (6 per tooth)) and the mean (GM-CEJ) (sum of all values divided by the number of sites examined (2 per tooth)).

The Bleeding On Probing (BOP) index evaluated the buccal, lingual, mesial and distal sulci of all teeth recommended by the World Health Organization based on the tendency to bleed after a standard stimulus and indicating periodontal inflammation. The four surfaces of each tooth were tested to provide a maximum total of 128 sites. A final index was obtained centrally and expressed as a percentage of sites positive for bleeding on probing. The BOP index was performed before the patient had received heparin administration for dialysis treatment.

3. Mucosal examination

The dental surgeon asked the participant about symptoms of oral pain, burning and dysgeusia (altered sense of taste). For pain and burning symptoms the patient indicated the severity of symptoms on a Visual Analog Scale reporting a value from 1 to 10. The dental surgeon also evaluated the presence of ulceration, white or red mucosal lesions, neoformation, petechial-ecchymosis, thrush, herpes, geographical tongue, scrotal tongue, uremic breath, previous surgery, and other lesions, indicating the oral location, when present. Drug-related gingival overgrowth (hypertrophy) was assessed as present or absent.

4. Salivary indices

The participant was then asked not to eat, drink, or smoke for an hour before the salivary analyses were performed. To estimate the salivary flow rate, the patient chewed on a standard weight (1 gram) of paraffin wax for 60 seconds and swallow the formed saliva. Then, while chewing the paraffin intensively, the saliva was spat into plastic sterile calibrated containers (5 centimeters in diameter and provided with a plastic cap) during the following 5 minutes. The stimulated salivary flow rate was expressed in milliliters per minute and measured before (pre-dialysis) and after dialysis (post-dialysis). The saliva was then used to measure the salivary buffering capacity using the CRT buffer system and salivary pH was determined using the Merck Universal indicator system (Merck, Darmstadt, Germany).

### Clinical follow-up and data collection

During follow-up, patients continued to receive usual standardized care according to dialysis center protocols at the entire discretion of the treating clinical team.

Data for total and cause-specific hospitalization and mortality will be obtained through data linkages to a centralized database within the Diaverum network. In this database, every change in patient status in the clinics is updated by the managing clinician on a monthly basis, including change of survival status or from present in clinic or hospitalized, with causes for death and hospitalization and days of hospital stay.

A cardiovascular-related death or hospitalization includes death or hospitalization attributed to acute myocardial infarction, pericarditis, atherosclerotic heart disease, cardiomyopathy, cardiac arrhythmia cardiac arrest, valvular heart disease, pulmonary edema, congestive cardiac failure, cerebrovascular accident including intracranial hemorrhage, ischemic brain damage including anoxic encephalopathy, or mesenteric infarction or ischemia of bowel. An infection-related death includes septicemia due to internal vascular access, central nervous system infection (brain abscess, meningitis, encephalitis), septicemia due to peripheral vascular disease or gangrene, or other cause, cardiac infection (endocarditis), pulmonary infection (pneumonia or influenza), abdominal infection (peritonitis, perforated bowel, diverticular disease, gallbladder), or genito-urinary infection (urinary tract infection, pyelonephritis, renal abscess).

### Outcomes

The primary outcome for the study is total (all-cause) mortality associated with different types of oral disease (such as edentulism, DMFT score, or periodontitis). Secondary outcomes include death attributable to cardiovascular causes and death due to infection. Additional secondary outcomes include total and cause-specific hospitalization (cardiovascular or infection), and prevalence and characteristics of oral diseases (edentulism, DMFT score, periodontitis, oral pain, dysgeusia, precancerous and cancerous lesions, salivary flow rates and saliva buffering capacity). Prevalence of oral hygiene habits, thirst, and dysgeusia will also be included in secondary analyses.

### Rationale for the number of participants

The sample size for this study is 4700 participants. Assuming that the prevalence of the exposure (i.e. periodontitis) is about 40% and that 1-year mortality in the exposed group is 15%, an overall sample of 4700 individuals (2820 with periodontitis and 1880 without) is sufficient to detect a relative reduction of at least 20% in 1-year mortality (i.e. absolute risk difference of 3%; odds ratio equal to 0.77) with a power of 80%, where correlation between exposure and other adjustment covariates = 0.10 and type I error = 0.05.

The large number of participants have also been recruited so that relatively rare conditions such as precancerous lesions of the mouth/tongue (estimated likely prevalence 1% to 3% [21]) can be evaluated. Recruitment has been competitive within the dialysis network to ensure that at least 4700 valid analyses with complete datasets and visits are available.

### Statistical analysis

Analysis will conform to a predefined statistical analysis plan agreed between the steering committee and study statisticians before analysis is commenced. Baseline sociodemographic and clinical characteristics will be calculated and expressed as mean (standard deviation) or median (25^th^ to 75^th^ percentile) for continuous variables and number (proportion) for categorical variables. Prevalence and characteristics of oral disease (dental, periodontal, mucosal, and salivary) will be calculated as number (proportion) affected for categorical variables and mean (standard deviation) or median (25^th^ to 75^th^ percentile) for continuous variables. Multivariable logistic regression models will be built to investigate the influence of patient demographic and clinical characteristics on risks of oral disease. We will include as potential variables in analysis: age, sex, ethnicity, country or residence, educational status, living situation, occupation status, smoking, BMI, self-reported appetite, income, financial strain, comorbidity score, diabetes, existing cardiovascular disease, depression score, blood pressure, protein catabolic rate, hemoglobin, albumin, phosphorus, cholesterol, ferritin, parathyroid hormone, and duration on dialysis.

We will use Cox proportional hazard models for analysis of mortality and hospitalization outcomes. We will check for confounders, interactions and multicollinearity among independent variables. The final models will be adjusted for all confounders and baseline covariables judged to have clinical importance. The significance level will be set at 0.05. All analyses will be performed using macro routines written in SAS Language (Release 9.1, SAS Institute Inc., Cary, NC; 2002–2003; http://www.sas.com/).

## Discussion

Chronic kidney disease is an important and increasing global public health problem that is associated with marked loss of quality of life and increased premature cardiovascular disease and mortality. End-stage kidney disease, the most severe form of kidney disease, is associated with profoundly reduced survival and a heavy symptom burden, although existing interventions to improve clinical outcomes in this population are generally lacking. Oral disease may represent an important cause of impaired quality of life and worse clinical outcomes in dialysis patients. However, on the basis of current evidence, data for the characteristics of oral health and preventative oral hygiene habits in dialysis population are sparse. Additional large-scale studies of oral health in this population would assist the prioritization of future research including appropriate interventional trials. The ORAL-D study is the first large and comprehensive survey of oral disease in adults treated with long-term hemodialysis. We will evaluate the prevalence, severity and correlates of oral disease and evaluate the relationship between oral disease and clinical outcomes (hospitalization and mortality) in this population.

## Abbreviations

BOP: Bleeding on Probing; CAL: Clinical Attachment Loss; CRT: Caries Risk Test; CEJ: Cementum-Enamel Junction; DMFT index: Decayed, Missing, Filled Teeth index; ICH guidelines: International Conference of Harmonisation guidelines; GM: Gingival Margin; ORAL-D study: The ORAL Diseases in hemodialysis study; PPD: Periodontal Probing Depth.

## Competing interests

The authors declare that they have no competing interests. SP receives an unrestricted fellowship from the Consorzio Mario Negri Sud from Amgen Dompé and is a L’Oreal For Women in Science Australia and New Zealand 2012 Fellow.

## Authors’ contributions

GFMS; Principal Investigator; the conception, design of the study, oversight of study conduct; SP; participated in design of the study, drafting of statistical analysis plan and initial drafting of manuscript; MR; participation in design of the study, oversight and conduct of all practical aspects of the study, critical review of the manuscript; PN; data collection and entry, critical review of the manuscript; VS, statistical analysis; JC, participation in design of the study, critical review of the manuscript; FP; statistical expertise; MP, MDB; participation in design of the study, oversight and conduct of all practical aspects of the study, critical review of the manuscript; PF, DJ; participation in design of the study, critical review of the manuscript; PS, LF, MT, MRL, EC, RG, AB-S, JD, JNF, DDC, JH, CW; participation in design of the study, oversight and conduct of practical aspects of the study, critical review of the manuscript. All authors have given final approval of the version to be published.

## Authors’ information

**Giovanni F.M. Strippoli**, MD, MPH, MM, PhD, Department of Clinical Pharmacology and Epidemiology, Consorzio Mario Negri Sud, Italy; Diaverum Scientific Office, Lund, Sweden; School of Public Health, University of Sydney, Australia, Department of Emergency and Organ Transplantation, University of Bari, Italy;

**Suetonia C. Palmer**, MBChB, PhD; University of Otago Christchurch, New Zealand

**Marinella Ruospo**, MSc; Diaverum Scientific Office, Lund, Sweden

**Patrizia Natale**, MSc; Diaverum Scientific Office, Lund, Sweden

**Valeria Saglimbene**, MSc; Department of Clinical Pharmacology and Epidemiology, Consorzio Mario Negri Sud, Italy

**Jonathan C. Craig**, MBChB, PhD; School of Public Health, University of Sydney, Australia

**Fabio Pellegrini**, MSc; Consorzio Mario Negri Sud; “Casa Sollievo della Sofferenza” Hospital, IRCCS, San Giovanni Rotondo (FG), Italy

**Massimo Petruzzi,** Dental clinic, University of Bari**,** Italy

**Michele De Benedittis,** Dental clinic, University of Bari, Italy

**Pauline J. Ford**, BDSc (Hons), BDentSt, PhD, GCHEd, The University of Queensland, School of Dentistry, Brisbane, Australia

**David W. Johnson**, MBBS (Hons), PhD, Centre for Kidney Disease Research, University of Queensland at Princess Alexandra Hospital, Brisbane, Australia

**Paul Stroumza**, MD, Diaverum Medical Office, France

**Luc Frantzen**, MD, Diaverum Medical Office, France

**Marietta Törok**, MD, Diaverum Medical Office, Hungary

**Miguel R Leal**, MD, Diaverum Medical Office, Portugal

**Eduardo Celia**, MD, Diaverum Medical Office, Argentina

**Ruben Gelfman**, MD, Diaverum Medical Office, Argentina

**Anna Bednarek-Skublewska**, MD, Diaverum Medical Office, Poland; and Department of Nephrology, Medical University School of Lublin, Poland

**Jan Dulawa**, MD, Diaverum Medical Office, Poland and Department of Internal Medicine, Metabolic Diseases, Medical University of Silesia, Poland

**Juan Nin Ferrari**, MD, Diaverum Medical Office, Uruguay

**Domingo Del Castillo**, MD, Diaverum Medical Office, Spain

**Jörgen Hegbrant**, MD, PhD, Diaverum Medical Office, Sweden

**Charlotta Wollheim**, MSc, Diaverum Medical Office, Sweden

Participating centers, facilitators, steering and organizing committee members

**Italy:** N Dambrosio, G Paparella, M Sambati, C Donatelli, F Pedone, VA Cagnazzo, R Antinoro, F Torsello, C Saturno, G Giannoccaro, S Maldera, E Boccia, M Mantuano, R Di Toro Mammarella, M Meconizzi, PF Steri, C Riccardi, A Flammini, L Moscardelli, M Murgo, N San Filippo, S Pagano, G Marino, G Montalto, S Cantarella, B Salamone, G Randazzo, D Rallo, A Maniscalco, M Fici, A Lupo, P Pellegrino, R Fichera, A D’Angelo, N Falsitta, L Gargano, P Natale

**Poland:** E. Bocheńska-Nowacka, A. Jaroszyński, J. Drabik, M. Birecka, D. Daniewska, M. Drobisz, K. Doskocz, G. Wyrwicz

**Portugal**: L Inchaustegui, C Outerelo, D Sousa Mendes, A Mendes, J Lopes, J Barbas, C Madeira, A Fortes, R Vizinho, A Cortesão, E Almeida, M Santos

**France**: C Boriceanu, S Frantzen-Trendel

**Hungary:** K Albert, I Csaszar, E Kiss, D Kosa, A Orosz, J Redl, L Kovacs, E Varga, M Szabo, K Magyar, G Kriza, E Zajko, A Bereczki, J Csikos, A Kuti, A Mike, K Steiner, E Nemeth, K Tolnai, A Toth, J Vinczene, Sz Szummer, E Tanyi, R Toth, M Szilvia, K Nagy, Ö Bajusz, I Pinke, G Decsi, L Gyergyoi, Zs Jobba, Zs Zalai, Á Zsedenyi, G Kiss, M Pinter, M Kereszturi

**Spain**: A. Bernat, B. De la Torre, A. Lopez, J. Martín , G. Cuesta, R.M. Rodriguez, F. Ros, M. Garcia, E. Orero, E. Ros.

**Argentina:** S Raña, M Serrano, S Claros, M Arias, L Petracci, M Arana, P De Rosa, A Gutierrez, M Simon, V Vergara, M Tosi, M Cernadas, I Vilamajó, D Gravac, M Paulón, L Penayo, G Carrizo, M Ghiani, G Perez, O Da Cruz, D Galarce, M Gravielle, E Vescovo, R Paparone, C Mato Mira, E Mojico, O Hermida, D Florio, M Yucoswky, W Labonia, D Rubio, G Di Napoli, A Fernandez, H Altman, J Rodriguez, S Serrano, G Valle, M Lobos, V Acosta, G Corpacci, M Jofre, L Gianoni, G Chiesura, M Capdevila, J Montenegro, J Bequi, J Dayer, A Gómez, C Calderón, E Abrego, C Cechín, J García, J Corral, M Natiello, A Coronel, M Muñiz, V Muñiz, A Bonelli, F Sanchez, S Maestre, S Olivera, M Camargo, V Avalos, E Geandet, M Canteli, A Escobar, E Sena, S Tirado, A Peñalba, G Neme, M Cisneros, R Oliszewski, V Nascar, M Daud, S Mansilla, A Paredes Álvarez, L Gamín, M Arijón, M Coombes, M Zapata

Doctors of Dental Surgery.

M Petruzzi (Dental clinic, University of Bari, Italy), M De Benedittis (Dental clinic, University of Bari) J Szkutnik (Department of Functional Masticatory Disorders Medical University of Lublin); J Sieczkarek (Chair and Department of Periodontology Medical University of Lublin); M Garcia Gallart (independent dentist), C Mendieta (Periodontology Professor, University of Barcelona); A Capelo (Department of Estomatology, Centro Hospitalar Lisboa Norte, Lisboa); A Caetano, K MacGregor, S Silva Pinheiro, L Martins, D Leitão, C Izidoro (independent dentists); G Bava (Buenos Aires); A Bora (Caleta Olivia); H Gorena (Córdoba); T Calderón (Mendoza); R Dupuy, N Alonso (Tucumán); V Siciliano (Comodoro Rivadavia).

## Pre-publication history

The pre-publication history for this paper can be accessed here:

http://www.biomedcentral.com/1471-2369/14/90/prepub
